# Regulation of *dgcZ* in EPEC E2348/69 and the effect of partially deleting its CZB domain on the type III secretion system

**DOI:** 10.1093/femsle/fnag040

**Published:** 2026-04-15

**Authors:** Alan S Rosette-Rueda, Haydee Martínez, José L Puente, Verónica I Martínez-Santos, Ricardo Oropeza

**Affiliations:** Department of Molecular Microbiology, Institute of Biotechnology, Universidad Nacional Autónoma de México, Cuernavaca, Morelos 62210, México; Department of Molecular Microbiology, Institute of Biotechnology, Universidad Nacional Autónoma de México, Cuernavaca, Morelos 62210, México; Department of Molecular Microbiology, Institute of Biotechnology, Universidad Nacional Autónoma de México, Cuernavaca, Morelos 62210, México; SECIHTI, Facultad de Ciencias Químico Biológicas, Universidad Autónoma de Guerrero, Chilpancingo, Guerrero 39090, México; Department of Molecular Microbiology, Institute of Biotechnology, Universidad Nacional Autónoma de México, Cuernavaca, Morelos 62210, México

**Keywords:** EPEC, LEE, DgcZ, BFP, virulence

## Abstract

Enteropathogenic *Escherichia coli* (EPEC) is a common cause of childhood diarrhea in developing countries. Extensive studies have established the role of virulence factors, such as the type III secretion system encoded by the locus of enterocyte effacement (LEE), in intestinal colonization and the central mechanisms that control their expression. However, the role of the second messenger c-di-GMP in virulence gene regulation in EPEC remains poorly understood, despite the extensive repertoire of genes encoding enzymes involved in its synthesis and degradation. In this work, we examined the regulation and role of DgcZ, a highly conserved diguanylate cyclase in commensal and pathogenic *E. coli* strains, in LEE gene regulation in the prototype EPEC strain E2348/69. Using reporter fusions of the *dgcZ* upstream regulatory region to the *cat* reporter gene, we first compared *dgcZ* promoter activity. A DgcZ deletion, partially lacking the CZB domain (DgcZ-∆NT), was constructed. Unexpectedly, the EPEC type III secretion system remains active when this deletion is introduced into EPEC growing under non-inducing conditions (LB medium). Whether this phenotype is caused by a protein-protein interaction between DgcZ lacking the CZB domain (DgcZ-∆NT) and an unknown effector remains to be determined.

## Teaser

DgcZ of EPEC appears to be regulated similarly to that of non-pathogenic *E. coli*; unexpectedly, virulence factors were detected when a truncated DgcZ was expressed in LB-grown bacteria.

## Introduction

Enteropathogenic *Escherichia coli* (EPEC) is one of the main pathogenic bacteria causing diarrhea in children worldwide, due to its high prevalence in communities (Kaur et al. 2023). EPEC belongs to the attaching and effacing (A/E) pathogen family, which attaches to the surface of intestinal enterocytes, causing lesions characterized by sloughing of intestinal epithelial microvilli, blunting of enterocyte brush borders, and the subsequent formation of an actin-rich pedestal-like structure beneath adherent bacteria (Gierynska et al. 2022, Miner et al. 2024).These bacteria carry a pathogenicity island called the locus of enterocyte effacement (LEE), which contains genes encoding the type III secretion system (T3SS), a syringe-shaped protein complex that EPEC uses to translocate >20 effector proteins into the host cell cytoplasm to induce A/E histopathology (Elliott et al. 1998, Serapio-Palacios et al. 2020). Additionally, classical EPEC strains carry the *bfp* operon on a plasmid known as the EPEC adhesion factor (EAF), which encodes bundle-forming pili (BFP) that aid in their initial adherence to the host cell (Hyland et al. 2008, Croxen et al. 2010).

Both non-LEE and LEE-encoded regulators control the expression of this 35-kb pathogenicity island (PAI), including Ler, H-NS, PerC, GrlA, and GrlR, which have been extensively studied (Platenkamp et al. 2018). H-NS, Ler, and PerC regulate the LEE; additionally, *per* also regulates the pEAF plasmid (Elliott et al. 2000). GrlA can independently activate *ler* expression and, as a result, LEE genes (Bustamante et al. 2011), while GrlR acts as a negative regulator by binding to GrlA (Lara-Ochoa et al. 2023).

The LEE region of EPEC strain E2348/69 contains 41 open reading frames (ORFs) organized into five major polycistronic operons (LEE1-5), two bicistronic operons (LEE6 and LEE7), and four transcriptional units (*etgA, cesF, map, and escD*) (Contreras et al. 2024). LEE encodes genes crucial for the T3SS, including regulators, chaperones, and effector proteins (Yerushalmi et al. 2014). These T3SS effector proteins affect host cell functions and promote bacterial survival and colonization. The components of the T3SS embedded in the inner and outer membranes (*esc* genes) are encoded by the LEE1, LEE2, and LEE3 operons (Elliott et al. 1998, Serapio-Palacios et al. 2020).

LEE regulation is controlled by several factors, with Ler, PerC, and GrlA serving as the key regulators of this island. The master regulator Ler, encoded by the first open reading frame (ORF) in LEE1, acts as a transcriptional anti-silencer of LEE2, LEE3, LEE4, and LEE5 by displacing the silencer H-NS and other nucleoid-associated proteins, thereby allowing transcription (Platenkamp and Mellies 2018).

PerC activates the LEE1 promoter, which encodes Ler, thereby stimulating the expression of virulence genes. Unlike Ler, PerC does not require H-NS for transcriptional activation; instead, it activates transcription through an unknown mechanism (Bustamante et al. 2011, Platenkamp and Mellies 2018).

On the other hand, the global regulator of Lee activator (GrlA) activates LEE1 transcription independently of PerC. Because GrlA and its own repressor, GrlR, are encoded in an operon within the PAI, Ler, in turn, increases GrlA expression in a positive feedback loop (Barba et al. 2005).

Although some T3SS proteins enable bacteria to adhere closely to epithelial cells, other factors, such as BfpA and EspA, can also contribute to bacterial clustering (Moreira et al. 2006).

It has been reported that different diguanylate cyclases (DGCs) and phosphodiesterases (PDEs) can produce distinct phenotypes, such as biofilm formation, indicating localized activity alongside specific target molecules. Furthermore, interactions between these proteins can act as a scaffold, creating local sources and sinks of c-di-GMP (Hengge et al. 2016). Additionally, c-di-GMP synthesis is involved in adhesion, motility, stress response, and biofilm development (Jayashree 2023 et al. 2023). Among the most conserved DGCs in *E. coli* pathovars is DgcZ, formerly known as YdeH. Its transcription in non-pathogenic *E. coli* is controlled by the two-component system CpxAR, which is activated by the lipoprotein NlpE in response to surface sensing (Otto et al. 2002).

It has been proposed that DgcZ integrates surface sensing, metabolism, and bacterial adhesion, coordinating environmental signals such as pH, nutrients, and oxidative stress with biofilm formation. This integration allows *E. coli* to adapt and stay attached to surfaces in changing environments, with both ecological and clinical implications, including chronic biofilm-associated infections (Lacanna et al. 2016).

In *E. coli* K-12, DgcZ contributes to the synthesis of the exopolysaccharide poly-β-1,6-N-acetylglucosamine (PGA) by producing c-di-GMP, which is essential for adhesion (Jayashree 2023 et al. 2023). Therefore, DgcZ regulates biofilm formation through post-translational control of PGA production (Boehm et al. 2009, Romling et al. 2013). This enzyme has a GGDEF catalytic domain fused to an N-terminal zinc-binding (CZB) domain spanning amino acids 22 to 89 (Zahringer et al. 2013). The CZB domain provides a homodimerization interface that brings the GGDEF domains together; however, when zinc is bound to this domain, it locks the catalytic domains of a homodimer into a nonproductive conformation (Gomelsky 2013). Dissection of CZB signal transduction shows that oxidation of the conserved zinc-binding cysteine controls CZB Zn^2+^ occupancy, which in turn regulates c-di-GMP synthesis by the associated GGDEF domains (Perkins et al. 2021).

In this work, the regulation of EPEC *dgcZ*, the biofilm-formation phenotype, and virulence factor expression were studied in the context of a partial deletion of the CZB domain of DgcZ.

## Material and methods

### Culture conditions

The strains used in this study are listed in Supplementary Table S1. They were cultured overnight at 37°C with shaking in 5 mL of LB medium (Luria-Bertani: 10 g/L casein tryptone, 5 g/L yeast extract, 10 g/L NaCl at pH 7.5). The next day, 50 µL of the overnight culture was inoculated into 5 mL of fresh LB, DMEM (Dulbecco’s Modified Eagle’s Medium) with 1% LB, or M9 minimal medium (200 mL of 5X M9 salt solution, 800 mL of distilled water, 2 mL of 1 M MgSO_4_ solution, 0.1 mL of 1 M CaCl_2_ solution, and 10 mL of 40% glucose) with the indicated antibiotics at the following concentrations: Sm 100 µg/mL, Tc 15 µg/mL, Ap 100 µg/mL, Km 30 µg/mL, and Gm 10 µg/mL. For solid media, 1.5% agar was added.

To explore the regulation by CysB and CpxR on pKK232-*dgcZb* and pKK232-*dgcZd*, experiments were conducted using different salt supplements: CuSO_4_ (2 mM), CuCl_2_ (2 mM), Na_2_SO_4_ (2 mM), and 0.2% cysteine.

### DNA extraction

For genomic DNA extraction, the Wizard^®^ Genomic DNA Purification Kit (Promega Corporation) was used, and for plasmid DNA extraction, the Zyppy™ Plasmid Miniprep Kit (Zymo Research) was used.

### PCR conditions

Amplification of genes or DNA fragments for cloning and/or sequencing was performed using polymerase chain reaction (PCR) at an annealing temperature of 56°C. Each reaction included the following components: template DNA (16 ng/µL), Forward and Reverse primers (20 pmol/µL each), dNTPs (1 mM), MgCl_2_ (3 mM), Buffer (1X), H_2_O, and Taq DNA polymerase (1 U/µL).

Samples from genomic and plasmid extractions, enzymatic digestions, and PCR products were analyzed using 1.5% agarose gel electrophoresis in 1X TAE buffer at 100 V and 40 mA for 45 min. The gel was stained with a diluted ethidium bromide solution for 5 min, rinsed with MilliQ water for another 5 min, and then visualized under ultraviolet light.

### Construction of *dgcZ* fusions in pKK232-8

The upstream sequence of the *dgcZ* gene was obtained from the *E. coli* O127:H6 E2348/69 (EPEC) genome database at www.genome.jp. Oligonucleotides flanking different parts of this region were designed, each containing a BamHI restriction site at the 5′ end and a HindIII restriction site at the 3′ end (Supplementary Table S2).

To clone the regions 5′ to the reading frame of the *dgcZ* gene, we used the plasmid pKK232-8 Tc, which contains an insert conferring tetracycline resistance between the BamHI and HindIII restriction sites (Thermo Fisher Scientific) and also confers ampicillin resistance. After amplifying the fragments for cloning, we ran them on a 1.5% agarose gel and purified the bands using the Zymoclean™ Gel DNA Recovery Kit. Following digestion, ligation reactions were set up and transformed into electrocompetent *E. coli* MC4100. Clones were selected on ampicillin plates, tested for tetracycline sensitivity, and the resulting plasmids were purified and sequenced (Supplementary Fig. S2).

### Cloning of *dgcZ* in pMPM-T6

The entire *dgcZ* ORF was cloned into the pMPM-T6 plasmid to generate pMPM-T6-*dgcZ* (Supplementary Table S1). The corresponding PCR product and the vector were digested with NcoI (Fast Digest, Thermo Fisher Scientific) followed by digestion with SalI (Thermo Fisher Scientific).

In the case of pMPM T6-*dgcZ* NdeI, which encodes the modified *dgcZ* gene without the sequence from codons 2–81 (Supplementary Fig. S1) and thus lacks the amino-terminal domain, it was constructed by digesting pMPM T6-*dgcZ* with NdeI (Thermo Fisher Scientific), an internal site in *dgcZ*, and then with NcoI (Fast Digest-Thermo Fisher Scientific).

The NcoI-NdeI-digested plasmid was repaired using the Basic Protocol for Repairing 3′ or 5′ Overhanging Ends (New England Biolabs) to generate blunt ends, and the plasmid was purified. The vector was self-ligated using 50 ng of vector, 1 µL of 10X Buffer, and 1 µL of T4 DNA ligase (1 U/µL). Both plasmids contain *dgcZ* ORFs under the control of an arabinose-inducible promoter, and the vector confers tetracycline resistance.

The ligation reactions were incubated overnight at 4°C. Electrocompetent *E. coli* MC4100 cells were transformed and selected on LB plates containing tetracycline. Plasmid DNA was extracted for sequencing, which was performed by the DNA Synthesis and Sequencing Unit at IBT UNAM. Once the plasmids were confirmed to have no mutations, EPEC E2348/69 cells were transformed with each construct.

### Chloramphenicol acetyltransferase assay

**Figure 1:**
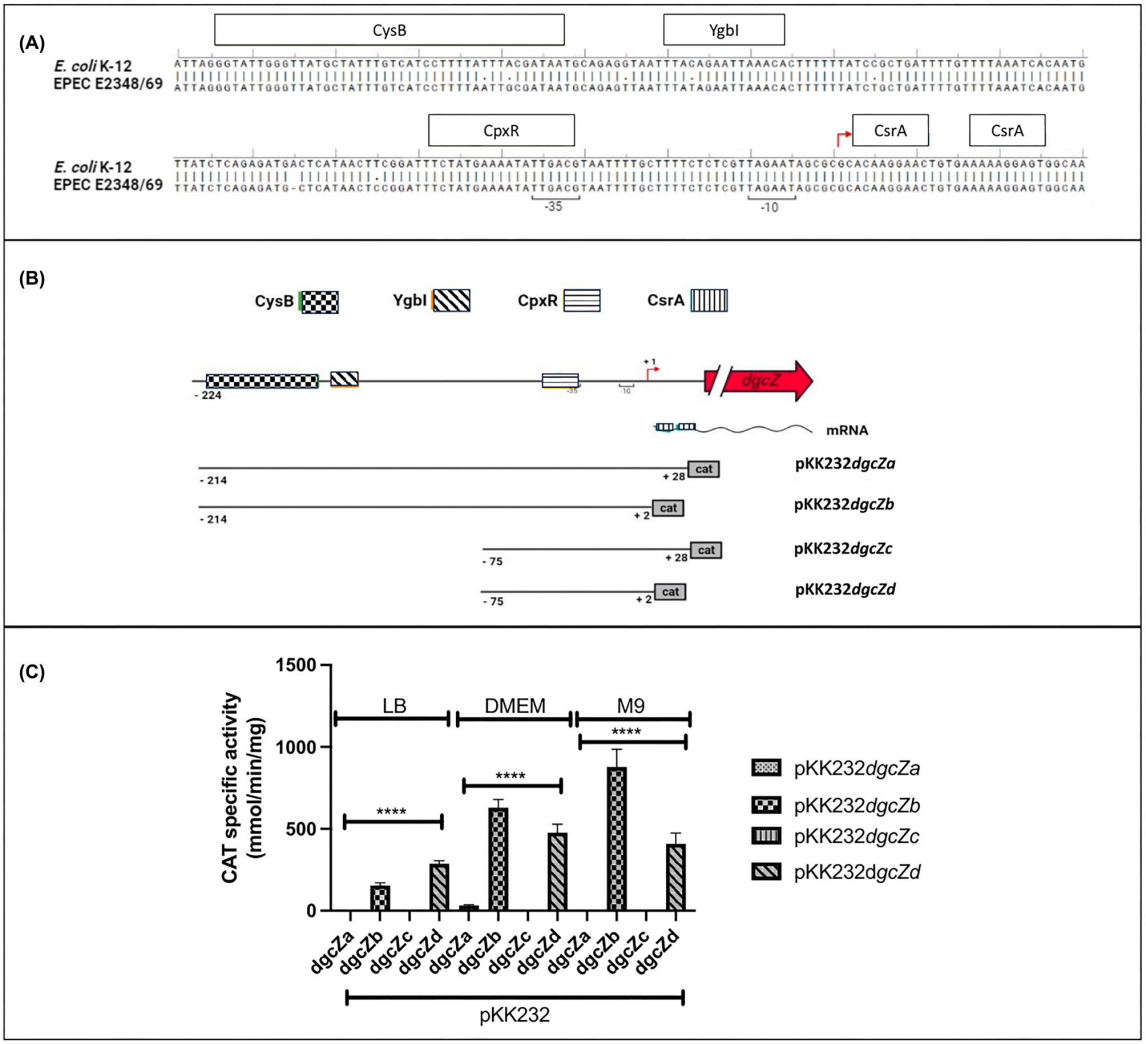
Demonstrable differences in activity in transcriptional fusions pKK232-*dgcZb* and pKK232-*dgcZd*. (A) Alignment of *dgcZ* regulatory sequences in *E. coli* K-12 and EPEC E2348/69. According to the alignment, the two sequences share 98.7% homology. A potential YgbI-binding box (as reported in previous work in our laboratory) is located downstream of the proposed CysB-binding box. (B) Schematic representation of transcriptional fusions of the EPEC *dgcZ* regulatory sequence to CAT. The numbers indicate the positions relative to the transcription start site. (C) Specific activity of transcriptional fusions in different media at OD_595_ of 0.8. EPEC transformed with pKK232-*dgcZa*, pKK232-*dgcZb*, pKK232-*dgcZc*, or pKK232-*dgcZd* was grown in different media (LB, DMEM, M9). Cultures were performed at 37°C with agitation. Data are presented as the mean of three independent experiments. ANOVA; ns, not significant (*P* > 0.05), ^∗^*P* < 0.05, ^∗∗^*P* < 0.01, ^∗∗∗^*P* < 0.001, ^∗∗∗∗^*P* < 0.0001.

The strain E2348/69 was transformed with fusions of *dgcZ* upstream sequences to chloramphenicol acetyltransferase (CAT) on the pKK232-8 Tc plasmid: pKK232-dgcZa, pKK232-dgcZb, pKK232-dgcZc, and pKK232-dgcZd (Fig. 1B). To analyze *LEE* or *bfpA*, EPEC was transformed with pKK232-8 *LEE1*-cat, pKK232-8 *LEE2*-cat, and pKK232-8 *bfpA*-cat fusions.

All strains were grown overnight in 5 mL of LB medium supplemented with Ap at 37°C with shaking (220 rpm). A total of 5 µL of the pre-inoculum was subcultured into 5 mL of LB medium supplemented with Ap and incubated at 37°C with shaking. One-milliliter samples were taken at OD_595_ = 0.6, 0.8, and 1.0. Each bacterium was inoculated in triplicate.

Samples were centrifuged at 12,000 rpm for 2 min. The supernatant was discarded, and the sample was washed twice with 800 µl of TDTT (Tris-dithiothreitol) and resuspended by vortexing. The sample was centrifuged again, and the pellet was resuspended in 600 µl of TDTT and vortexed. The resuspended pellet was sonicated, then centrifuged at 12 000 rpm for 15 min, and the soluble extract was transferred to new tubes.

The CAT assay was conducted as previously described (Puente et al. 1996, Bustamante et al. 2001). Briefly, soluble extracts were added to a microtiter plate. Changes in absorbance at 410 nm were monitored for 5 min after adding a mixture containing 1 mM DTNB [5,5′-dithiobis(2-nitrobenzoic acid)] and 0.1 mM acetyl-coenzyme A (acetyl-CoA). Protein concentration was measured using the BCA Protein Assay Kit. Briefly, 10 μL of protein extract was added to a 96-well plate in duplicate, and 200 μL of the reaction mixture (composed of 25 mL of solution A and 500 μL of solution B) from the BCA Protein Assay Kit (Pierce) was added to each sample. After incubation at 37°C for 30 min, the 96-well plate was read in the automatic microplate reader CERES 900 C.

CAT-specific activity (expressed as µmol/min/mg of protein) was subsequently calculated.

### Protein secretion assay

From overnight cultures, 1:100 dilutions were prepared in LB or DMEM containing 0.01% arabinose and incubated at 37°C with shaking until the cultures reached an OD_595_ nm of ~1.0. Then, 4.5 mL of culture was centrifuged at 14 000 rpm for 5 min. Subsequently, 720 µL of supernatant was transferred to a new tube (the pellets were stored at −20°C for use in the Western blot assay), and protein precipitation was carried out using MeOH/chloroform. Alcohol precipitation involved adding 540 µL of methanol and vortexing, followed by the addition of 180 µL of chloroform and the mixture was vortexed. The samples were then centrifuged at 4°C for 5 min at 14 000 rpm. The supernatant was carefully removed without disturbing the interface between the phases. Next, 540 µL of methanol was added and vortexed, and the mixture was centrifuged again at 14 000 rpm for 10 min at 4°C. The supernatant was removed, and the pellet was left to dry. The pellet was resuspended in 25 µL of protein loading buffer (composed of 5 mL 100% glycerol, 2 mL 20% SDS, 1 mL 10% v/v β-mercaptoethanol, 2.5 mL 1 M Tris–HCl pH 6.8, and 0.02 g bromophenol blue) and heated at 95°C for 10 min. The samples were then analyzed by 12% sodium dodecyl sulfate polyacrylamide gel electrophoresis (SDS-PAGE). After electrophoresis, the gel was stained with Coomassie blue and destained using a 0.2% acetic acid-methanol solution.

### Western blot assay

The bacterial pellets from the protein secretion assay were resuspended in 300 µL of 1X PBS and sonicated. An amount of 100 µL of 4X Laemmli Loading Buffer was added, and the pellets were heated at 95°C for 10 min. Each sample was homogenized and analyzed by 12% SDS-PAGE. The proteins were transferred to a nitrocellulose membrane (Millipore) using a semidry transfer chamber (Bio-Rad). The membrane was blocked with PBS-T (100 mL PBS 10X + 100 mL 5 M NaCl + 1 mL Tween 20 + 800 mL H_2_O) mixed with 5% non-fat milk, washed with PBS-T, and incubated with the following primary antibodies to detect virulence factors encoded in LEE, such as α-Tir, α-EspB, and α-EscJ. The α-GroEL chaperone was used as a loading control (antibodies were kindly provided by the laboratory of Dr. Bertha González-Pedrajo). Antibodies were prepared at 1:50 000 for rabbit polyclonal anti-GroEL (Sigma–Aldrich), and 1:10 000 for α-Tir, 1:10 000 for α-EspB, and 1:10 000 for α-EscJ.

The membranes were rewashed and incubated with a 1:10 000 dilution of horseradish peroxidase-conjugated anti-rabbit antibody (Rockland). They were developed using the Western Lightning Plus-Enhanced Chemiluminescent Substrate (PerkinElmer) according to the manufacturer’s instructions. Bands were detected with X-OMAT LS films (Carestream, Sigma).

### Biofilm assay

Overnight cultures of the strain E2348/69 EPEC, carrying pMPM-T6, pMPMT6-*dgcZ*, and pMPM T6-*dgcZ* NdeI, were diluted 1:100 in 0.5X LB medium containing the appropriate antibiotic. Each culture was also supplemented with 0.1% arabinose for induction or left without it as a negative control.

Two hundred microliter of culture dilution was transferred in quadruplicate to each well of two 96-well polystyrene plates and incubated at 30°C or 37°C for 24 h. After incubation, total culture growth was determined by measuring OD at 620 nm. The culture was then discarded from the wells and washed three times with 200 µL of 1X PBS. The biofilm was fixed with 200 µL of 100% methanol for 15 min, then removed and allowed to dry for 10 min. Once dry, the plates were stained with 200 µL of 2% crystal violet for 10 min; the stain was then discarded. The plates were rinsed with water three times and dried at 55°C for 15 min. Finally, the dye was resolubilized by adding 200 µL of 33% acetic acid and shaking for 15 min; then, the optical density was measured at 570 nm for biofilm (procedure modified from (Stepanovic et al. 2000). Biofilm formation was calculated as the biofilm optical density divided by the total growth OD.

### Statistical analysis

To determine whether the data from the biofilm formation assays and reporter fusion expression were statistically significant, Welch’s *t*-test or ANOVA was performed on samples showing a phenotypic effect.

## Results

### Analysis of the transcriptional activity of *dgcZ-cat* fusions

To design the *dgcZ-cat* fusions, an alignment was performed between the *dgcZ* upstream sequence of *E. coli* K-12, as reported in the RegulonDB Database, and the corresponding sequence of EPEC E2348/69 to identify common regulatory elements (Fig. 1A). According to the database, the *dgcZ* gene in the non-pathogenic strain has three regulatory sites: a CysB-binding site, a CpxR-binding site, and two CsrA-binding sites (CsrA binds mRNA post-transcriptionally). The alignment shows 98.7% identity between the two sequences.

Furthermore, we investigated the potential effect of YgbI on a proposed box located downstream of the proposed CysB-binding site. The *ygbI* gene encodes an uncharacterized protein; sequence similarity studies indicate it is a transcriptional regulator of the DeoR family. Regulators in this family usually act as repressors in sugar metabolism, and often, the inducer is a phosphorylated sugar (as noted in previous work in our laboratory).

Taking this into account, four transcriptional fusions to CAT were constructed, as shown in Fig. 1B, to identify potential regulatory elements within the *dgcZ* upstream sequence. The activity of these fusions in EPEC E2348/69 was measured under different growth conditions.

To analyze the effect of the growth medium, EPEC carrying each transcriptional fusion was cultured in LB, DMEM, and M9 minimal medium at 37°C with shaking.

When comparing EPEC transformed with pKK232*-dgcZ*b or pKK232*-dgcZ*d to those containing the pKK232-*dgcZa* or pKK232-*dgcZc* plasmids, which include the CsrA-binding sequences, the latter showed little to no activity in any medium (Fig. 1C). This suggests that this post-transcriptional regulator binds to the *dgcZ* mRNA in EPEC.

In LB medium, the activity of the pKK232-dgcZb fusion, which contains the CysB and CpxR boxes (predicted sites), was lower than that of the pKK232-dgcZd fusion, which includes only the CpxR box. However, this activity profile changed when bacteria were grown in DMEM and M9 minimal medium (Fig. 1C). It has been reported that, in the presence of copper, the CpxA/R two-component system is activated because copper can affect the bacterial membrane (Yamamoto et al. 2006, Sakamoto et al. 2012). Furthermore, CysB is known to induce transcription of genes involved in cysteine biosynthesis and sulfate metabolism in *E. coli* when these compounds are present at low concentrations, at least in M9 minimal medium (Kawano et al. 2015).

**Figure 2:**
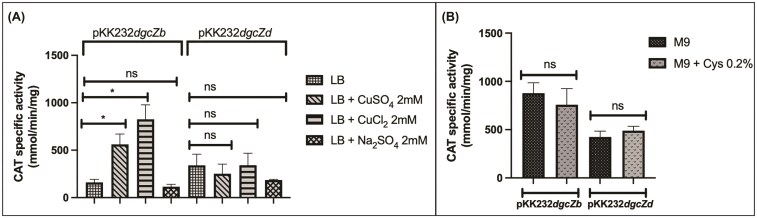
Copper, but not cysteine, increases the activity of the pKK232-*dgcZb* fusion but not that of the pKK232-*dgcZd* fusion. (A) CAT specific activity of the pKK232-*dgcZb* and pKK232-*dgcZd* transcriptional fusions in LB supplemented with 2 mM of salts CuSO_4_, CuCl_2_, and Na_2_SO_4_ (OD_595_ = 0.8). (B) CAT specific activity of the pKK232-*dgcZb* and pKK232-*dgcZ* fusions in M9 minimal medium supplemented with 0.2% cysteine (OD_595_ = 0.8). Data are presented as the mean of three independent experiments. Welch’s test; ns, not significant (*P* > 0.05), ^∗^*P* < 0.05, ^∗∗^*P* < 0.01, ^∗∗∗^*P* < 0.001, ^∗∗∗∗^*P* < 0.0001.

The expression of the two activated fusions was evaluated in LB medium with different salts (Fig. 2A). EPEC transformed with pKK232-*dgcZb* showed increased activity in LB medium with either CuSO_4_ or CuCl_2_, with CuCl_2_ having the strongest effect. In contrast, supplementing LB with Na_2_SO_4_ did not significantly change the specific activity. Conversely, EPEC carrying pKK232*dgcZd* showed no significant change under any of the conditions tested (Fig. 2A).

Adding copper salts to LB increased the activity of the pKK232-*dgcZb* fusion. However, this increase seems to depend on the presence of the full regulatory sequence, as the fusion containing only the CpxR-binding box was not affected by copper in the medium. Additionally, sulfate had no effect on the specific activity of either fusion (Fig. 2A).

To evaluate the potential effect of CysB on the fusions, activity was measured in M9 minimal medium supplemented with cysteine (Fig. 2B). The addition of cysteine did not influence the activity of any of the transcriptional fusions.

**Figure 3:**
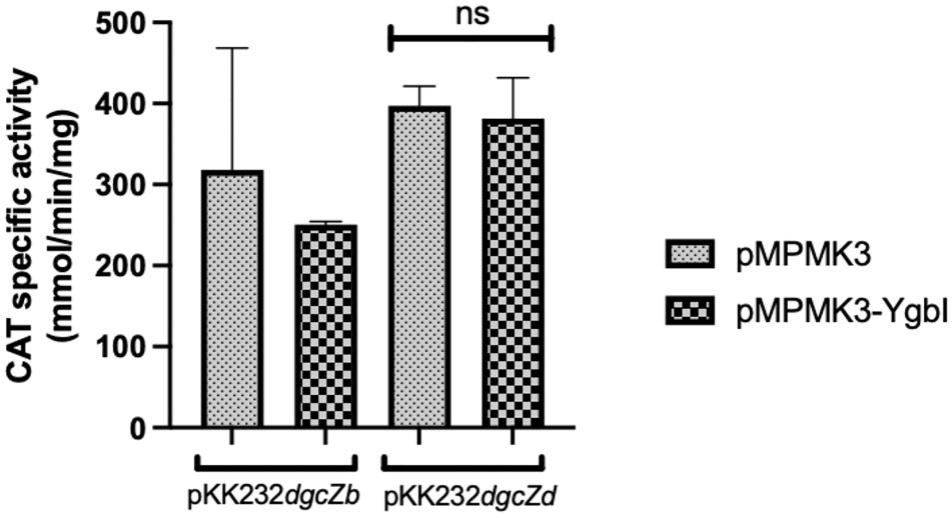
YgbI induction did not significantly change the expression of transcriptional fusions pKK232-*dgcZb* and pKK232-*dgcZd*. CAT specific activity of the pKK232-*dgcZb* and pKK232-*dgcZd* fusions was measured when the YgbI protein was induced (OD_595_ = 0.8). Welch’s test; ns, not significant (*P* > 0.05), ^∗^*P* < 0.05, ^∗∗^*P* < 0.01, ^∗∗∗^*P* < 0.001, ^∗∗∗∗^*P* < 0.0001.

We tested whether the induction of the YgbI protein (a putative transcriptional repressor involved in biofilm formation) affected the activity of the pKK232-*dgcZb* fusion, which includes a putative YgbI-binding box. The pKK232-*dgcZd* fusion was used as a control because it lacks this binding site; the empty plasmid pMPMK3, used for *ygbI* cloning, was included as well. As shown in Fig. 3, EPEC transformed with pKK232-*dgcZb* showed no change in activity upon YgbI induction, and the same result was observed with the empty vector. This experiment indicates that under these conditions, YgbI does not regulate the promoter, despite the presence of its predicted binding site (Fig. 3).

### Assessment of the effect of expressing DgcZ without the CZB domain on biofilm formation and virulence factors

Because DgcZ is a diguanylate cyclase conserved across different *E. coli* strains, it has only been characterized in the non-pathogenic strain and is linked to biofilm formation. Therefore, biofilm formation was analyzed.

Biofilm formation in EPEC was assessed in LB medium diluted 1:1 with water at 30°C and 37°C for 24 h. The empty plasmid pMPM-T6 (Supplementary Table S1) was used as a negative control. Additionally, the effect of inducing full-length *dgcZ* was compared with that of *dgcZ* NdeI (*dgcZ* lacking its CZB N-terminal domain, DgcZ-∆NT). As shown in the results (Supplementary Fig. S2), there was no increase in biofilm formation when *dgcZ* or *dgcZ* without the CZB domain was induced in EPEC relative to the negative control during the first 24 h at 30°C.

Similarly, when the plate was incubated at 37°C for the same time (Supplementary Fig. S3), no increase in biofilm formation was observed. From this, we can conclude that neither the full-length DgcZ protein nor a partial deletion of its N-terminal domain promotes biofilm formation.

### Expression of the C-terminal domain of DgcZ, but not the wild-type protein, induces LEE transcription under repressive conditions

To evaluate the effect of DgcZ on the transcription of selected EPEC virulence genes, the activity of the previously constructed fusions pCAT232-8 *bfpA-cat*, pKK232-8 *LEE1*-cat, and pKK232-8 *LEE2*-cat was measured in cultures of EPEC E2348/69 carrying these fusions, grown in LB medium, which is a condition known to be non-permissive for the expression of the LEE and other virulence factors (Lara-Ochoa et al. 2023).

**Figure 4:**
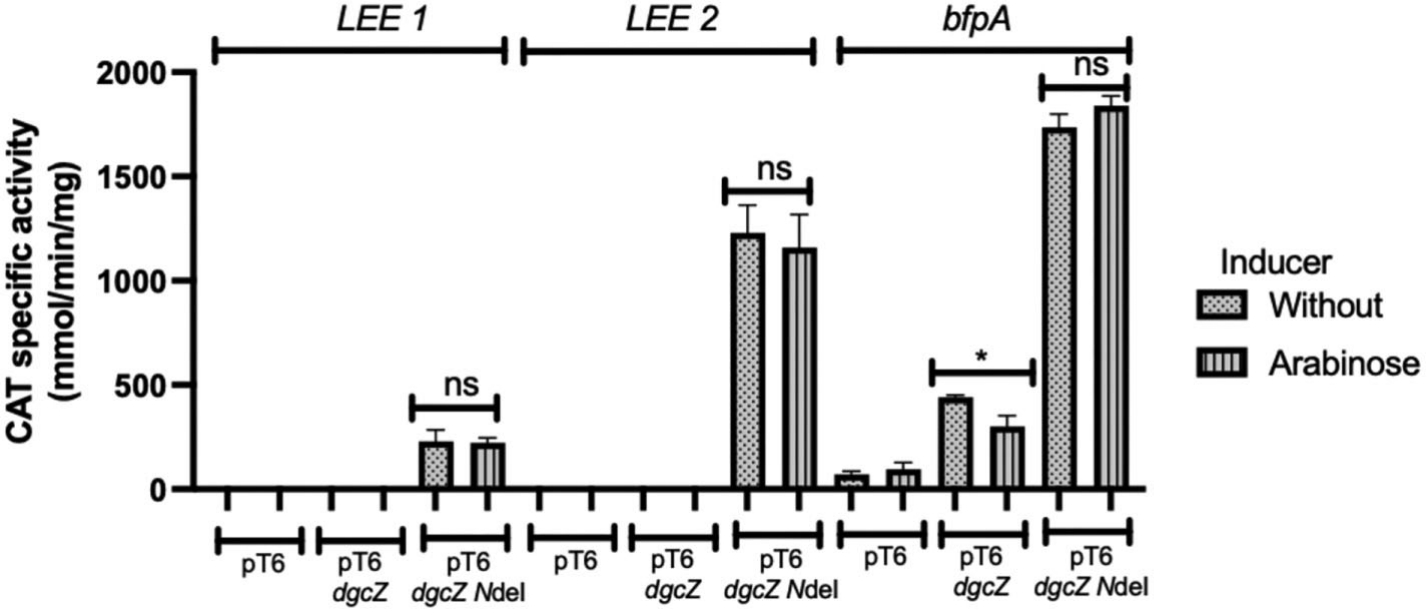
DgcZ-∆NT induces *the expression of LEE 1, LEE 2*, and *bfpA* with and without arabinose. CAT-specific activity of the pKK232-8 *LEE1*-cat fusion, pKK232-8 *LEE2*, and pKK232-8 *bfpA* in EPEC WT transformed with the indicated pT6 plasmids in LB medium. Cultures were grown on LB or DMEM. ANOVA; ns, not significant (*P* > 0.05), ^∗^*P* < 0.05, ^∗∗^*P* < 0.01, ^∗∗∗^*P* < 0.001, ^∗∗∗∗^*P* < 0.0001.

No activity was observed in the wild-type EPEC strain carrying plasmids pMPM-T6 or pMPM-T6-*dgcZ* for any of the fusions, regardless of the presence of the inducer (Fig. 4). However, in the presence of plasmid pMPM-T6-*dgcZ* NdeI (DgcZ-∆NT), expression of all three fusions was significantly increased with or without arabinose induction, indicating that basal transcription from the pBAD promoter was sufficient to activate the transcriptional fusions.

**Figure 5:**
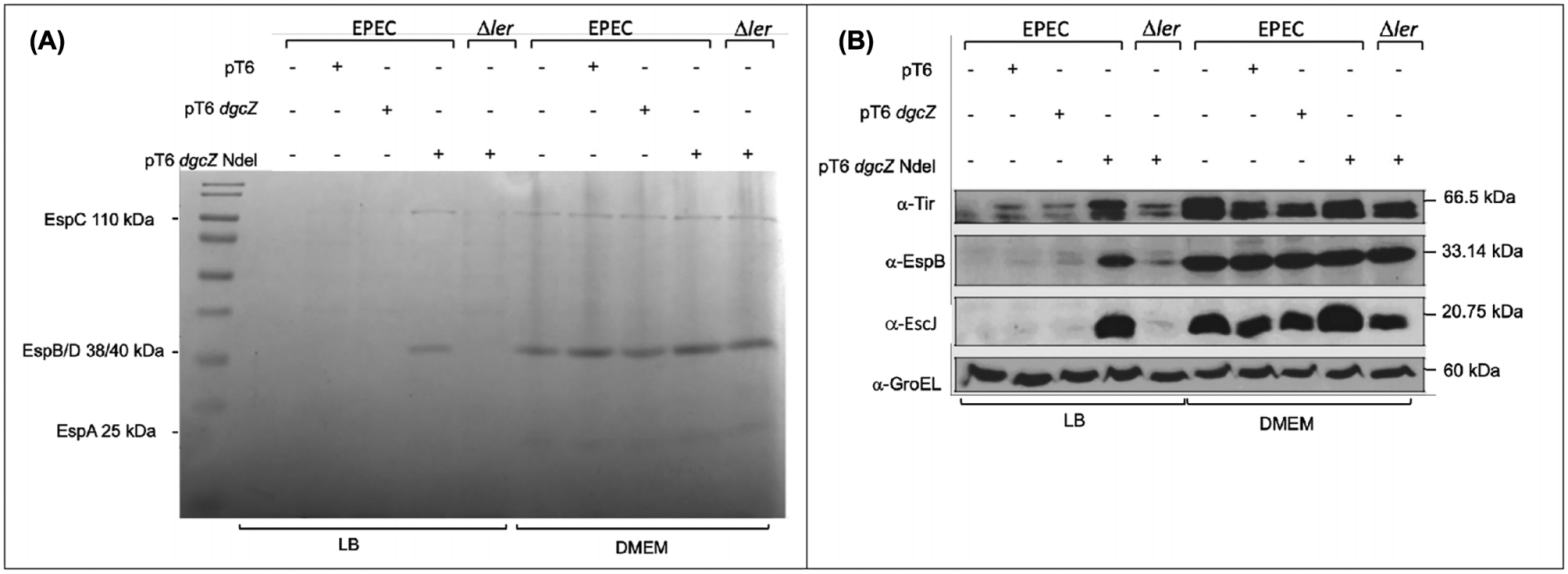
DgcZ-∆NT regulates LEE expression through Ler in LB. (A) Profile of secreted proteins by EPEC strains wt and Δ*ler* transformed with pMPMT6, pMPMT6 dgcZ, or pMPMT6 dgcZ Ndel, grown in LB and DMEM media, induced with 0.01% arabinose, and incubated at 37°C with agitation (samples were taken at OD_595_ = 1). (B) Western blot of cell lysates from the cultures used in panel A.

To confirm this phenotype and assess its effect on T3SS function, culture supernatants were used for secretion assays, and whole-cell extracts were analyzed by Western blot to detect LEE-encoded translocators and effector proteins under both LEE-inducing (DMEM) and non-inducing (LB) growth conditions. Expression of DgcZ-∆NT, but not full-length DgcZ, in the wild-type strain promoted secretion (Fig. 5A) and expression (Fig. 5B) of LEE-encoded proteins under non-permissive conditions (LB). No differences were observed in DMEM cultures, where LEE expression is known to be derepressed. Importantly, DgcZ-∆NT did not restore LEE gene expression in the EPEC ∆*ler* grown in LB, but it did in DMEM (Supplementary Fig. S4).

To analyze the pathway activated by DgcZ NdeI (DgcZ-∆NT) that induces LEE expression, this protein was expressed in different genetic backgrounds carrying deletions of the most important regulators of this pathogenicity island in EPEC, as well as in an EPEC strain lacking the EAF plasmid.

**Figure 6:**
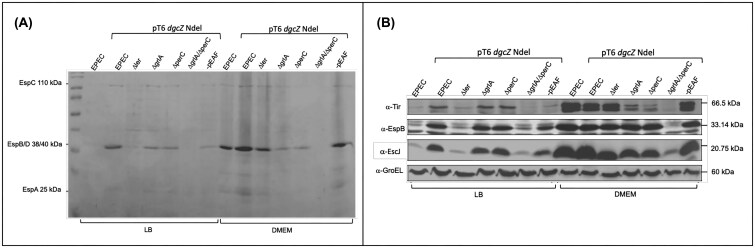
Both Ler and DgcZ-ΔNT regulate LEE expression, even in the absence of GrlA and PerC. (A) Profile of secreted proteins from EPEC strains Wt, Δ*ler*, Δ*grlA*, Δ*perC*, Δ*grlA*/Δ*perC*, and pEAF^−^ carrying pMPMT6 *dgcZ* Ndel, grown in LB and DMEM at 37°C with agitation (samples collected at OD_595_ = 1). Expression of DgcZ-ΔNT was induced with 0.01% arabinose. (B) Western blot of cell lysates from the cultures in panel A. GroEL was used as a loading control.

As shown in Fig. 6A, in LB medium, we continued to detect the proteins encoded by LEE in the *grlA* and *perC* mutants and in the EAF plasmid-cured strain, although less prominently than in the wild-type strain. It is known that GrlR and H-NS negatively regulate LEE gene expression under repressive conditions, such as growth in LB. Moreover, secretion was not restored in the *grlA*-*perC* double mutant.

In DMEM medium, most secretion profiles resembled those in LB medium, with reduced protein levels in the *grlA* and *perC* mutants and no protein secretion in the double mutant for these activators. Notably, the secreted protein profile of the strain cured of the EAF plasmid is more evident in this medium. To further support these findings, a Western blot assay was performed, inducing *dgcZ* NdeI (DgcZ-∆NT) in these mutants. As shown in Fig. 6B, the presence of these virulence factors is confirmed in total lysates of the *grlA* and *perC* mutants and in those lacking the EAF plasmid. Additionally, they were not detected in the double mutant for these activators; clearly, DgcZ NdeI (DgcZ-∆NT) activates LEE under non-permissive conditions. However, GrlA or PerC are also necessary for this activation.

**Figure 7:**
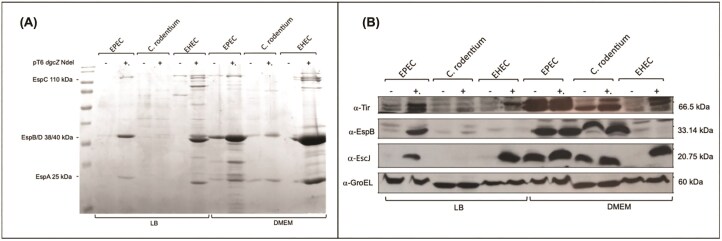
DgcZ-∆NT induces LEE expression in EHEC and EPEC, but not *C. rodentium* when grown in LB. (A) Secreted protein profile of A/E pathogens transformed with pMPMT6 *dgcZ* NdeI and grown in LB and DMEM at 37°C with agitation (samples taken at OD_595_ = 1). Expression of DgcZ-∆NT was induced with 0.01% arabinose. (B) Western blot of cell lysates from A/E pathogens used in panel A.

Protein secretion assays and Western blots were performed, inducing *dgcZ* NdeI (DgcZ-∆NT) in other A/E pathogens that possess the LEE. As shown in Fig. 7A and B, enterohemorrhagic *Escherichia coli* (EHEC) exhibits a secreted protein profile and Western blot detection similar to that observed in EPEC when this protein is induced in either LB or DMEM media.

In *C. rodentium, dgcZ* Ndel (DgcZ-∆NT) seems unable to induce the same phenotype as in EPEC and EHEC. This murine pathogen lacks the *dgcZ* and *perC* genes, which may explain this outcome. However, it has proteins that regulate LEE in a way similar to those in EPEC. Instead of PerC, it has RegA (Lara-Ochoa et al. 2021). Therefore, we suggest that *C. rodentium* is missing specific protein(s) needed for LEE activation when DgcZ lacks the CZB domain. Whether the effect of DgcZ-∆NT extends to other T3SSs remains to be determined.

## Discussion

EPEC is a pathogen that infects children under 5 years of age, causing morbidity and mortality. EPEC’s ability to colonize results from an organized process that includes microvilli adhesion and sloughing, facilitated by virulence factors, adhesion factors, and the T3SS. Similarly, its precisely coordinated signaling mechanism, which is sensitive to changes in nutrients and molecules, enables it to form a biofilm or shift from a sessile to a planktonic state. However, these processes are not fully understood.

In this context, it is important to note that c-di-GMP is produced by diguanylate cyclases, such as DgcZ, which is the focus of this study. Several pathotypes of *E. coli*, including EPEC and uropathogenic *Escherichia coli* (UPEC), have been shown to use c-di-GMP to regulate virulence factor expression (Hall et al. 2018).

Therefore, we analyzed the regulatory region of the gene encoding the diguanylate cyclase DgcZ in EPEC. This gene contains three regulatory sites: a CysB-binding site, a CpxR-binding site, and two CsrA-binding sites (CsrA binds post-transcriptionally to mRNA) (Fig. 1A and B).

CsrA typically binds to sites containing critical GGA motif(s) in the 5′ untranslated region (5′-UTR) and/or the early coding region of its messenger RNA (mRNA) targets, affecting RNA structure, translation, stability, and/or transcription elongation (Vakulskas et al. 2015). CsrA is a key component of the Csr (carbon storage regulator) system and plays a role in biofilm formation (Potts et al. 2017). Based on the results obtained (Fig. 1C), we suggest that CsrA binds to the EPEC *dgcZ* mRNA under the culture conditions used in this work, as reported for non-pathogenic *E. coli*.

Since fusions lacking the CsrA-binding site were active under the experimental growth conditions (Fig. 1C), these fusions were further analyzed to elucidate the regulatory region of EPEC dgcZ.

Regarding the CpxR region and the effect of copper salts, activity is observed in the pKK232*dgcZb* fusion but not in the shorter fusion, indicating that regulation extends beyond the proposed CpxR binding box. Notably, the fusion containing only the putative CpxR box does not respond to copper under these growth conditions (Fig. 2A). Additionally, it has been reported that the CpxA/R system and *dgcZ* in *E. coli* BW25113 respond to copper and favor biofilm formation (Price et al. 2009).

As previously reported, CysB promotes the transcription of genes involved in cysteine biosynthesis and sulfate metabolism in *E. coli* when these compounds are absent from the environment. In turn, CysB is inhibited by these reagents through a negative feedback loop (Kawano et al. 2015). The absence of CAT activity induction when bacteria are grown without these compounds suggests that more research is needed to determine whether CysB controls *dgcZ* in EPEC (Fig. 2B).

Likewise, YgbI does not regulate *dgcZ* fusion activity under these conditions (Fig. 3).

Interestingly, biofilm formation in EPEC was not induced by either the truncated or the full-length DgcZ compared to the empty vector in the presence of arabinose (Supplementary Figs S2 and S3).

It has been reported that residues 19–90 of DgcZ belong to the Pfam domain CZB (Zahringer et al. 2013). This domain features the highly conserved presence of three histidine residues and one cysteine that coordinate zinc ion binding in DgcZ. In the case of full-length DgcZ, zinc allosterically inhibits the enzyme by binding tightly to its CZB domain (Zahringer et al. 2013). Conversely, the truncated form of DgcZ (DgcZ-ΔNT) cannot dimerize or bind zinc because it lacks the CZB domain; therefore, it cannot produce c-di-GMP or support biofilm formation.

Furthermore, it has been reported that the zinc-depleted form of DgcZ, expressed in *E. coli* K12, promotes biofilm production that depends on the *pgaABCD* biosynthetic operon (Zahringer et al. 2013). A bioinformatic analysis of the EPEC E2348/69 genome to locate the *pga* locus identified an insertion/deletion difference between these two strains, which includes the *pga*ABCD operon. Therefore, EPEC cannot utilize the DgcZ-Pga pathway to form biofilm.

However, when the N-terminal domain of DgcZ is missing (DgcZ-∆NT), the remaining protein activates the EPEC T3SS encoded within the LEE pathogenicity island. This was demonstrated by the activation of operon reporter fusions, secretion of virulence factors, and detection of LEE-encoded proteins through Western blot (Figs 4 and 5A and B). Importantly, this phenotype results from a monomeric, inactive form of the diguanylate cyclase DgcZ (DgcZ-∆NT) (Zahringer et al. 2013).

Furthermore, this induction was also observed in EHEC in both LB and DMEM (Fig. 7). However, in EHEC, LEE activation has been reported to depend on the presence of 44 mM NaHCO_3_ in LB (Abe et al. 2002). In contrast, the growth conditions used in this study employ a bicarbonate-free LB medium. Therefore, LEE stimulation results from the presence of DgcZ lacking the CZB.

It is possible that during the intestinal infection by EHEC or EPEC, NaHCO₃ is released in the small or large intestine, which might stimulate LEE expression to enhance colonization. Determining whether this is due to DgcZ lacking the CZB domain or another unknown factor requires further investigation.

Additionally, evidence shows that LEE in EHEC is activated during the stationary growth phase. Therefore, virulence factors were not detected in DMEM at the OD used (even with bicarbonate present) in the wild-type strain; they were detected when *dgcZ* NdeI (DgcZ-∆NT) was induced (Fig. 7A and B). This suggests that inducing this protein may activate the EHEC pathogenicity island, regardless of whether the strain is under optimal conditions for its expression.

Thus, DgcZ-ΔNT may control LEE through an unidentified factor. In LB, the Δ*ler* strain expressing DgcZ-ΔNT does not promote LEE expression, as expected under repressive conditions. However, DMEM is considered an induction medium only with Ler regulation; in the absence of Ler, our results suggest that LEE regulation occurs independently of Ler but still requires DgcZ-ΔNT (Supplementary Fig. S4).

Our working model suggests that the DgcZ protein undergoes proteolytic cleavage, similar to other diguanylate cyclases (Perry et al. 2004, Herbst et al. 2018, Joshi et al. 2020). In this process, cleavage occurs between the CZB amino terminus and the diguanylate cyclase C-terminus. After cleavage, the truncated monomeric protein may regulate the expression of LEE and *bfpA*, as shown here. Notably, diguanylate cyclases require dimerization to be active. DgcZ-ΔNT likely depends on interaction with an unknown protein to activate BfpA and LEE genes in EPEC. Additionally, DgcZ-mediated activation of LEE could result from interactions between bacteria and eukaryotic cells, leading to c-di-GMP production (Jayashree 2023 et al. 2023). This process, in turn, activates proteases that mediate DgcZ activation through proteolysis, producing a monomer (DgcZ-ΔNT) that affects BfpA and LEE, potentially involving an unknown partner.

The expression and assembly of the type III secretion system encoded in the LEE pathogenicity island can occur under non-permissive culture conditions when DgcZ-∆NT is induced. This suggests that it interacts with another partner and indirectly regulates the LEE pathogenicity island. This occurs even when essential genes for LEE regulation are deleted, as shown in Fig. 6. It should be noted that DgcZ of EHEC and EPEC share 97.97% identity. Therefore, the activation mechanism of the LEE island is likely very similar between these two pathotypes. In this context, it would be important to evaluate other strains that harbor this pathogenicity island and *dgcZ*, such as *Escherichia albertii*, to determine whether the same result is obtained. Interestingly, *Citrobacter rodentium* harbors the LEE pathogenicity island but lacks *dgcZ*, which could explain the observed result for this strain.

## Conclusion

Based on the transcriptional fusion results, the CpxR-binding box and the integrity of the proposed CysB box in the *dgcZ* regulatory region are essential for its activation. Furthermore, the post-transcriptional regulator CsrA plays a significant role in controlling *dgcZ*, at least under the conditions tested in this study.

Since CsrA has been reported to bind the leader mRNAs of *dgcZ* and *grlAR*, CsrA binding decreases transcript levels (Bhatt et al. 2009). However, a counteracting factor could regulate LEE expression.

The DgcZ protein, with or without a partial deletion of its CZB domain (DgcZ-∆NT), does not affect biofilm formation in EPEC under the tested conditions. However, DgcZ lacking the CZB domain (DgcZ-∆NT) activates various virulence factors, such as the LEE and BFP, through an unknown pathway.

This activation is independent of the presence of *grlA* or *perC* under both LEE non-permissive conditions (LB medium) and activation conditions (DMEM medium), but requires at least one of them. It does not occur in the absence of *ler* in LB, but is independent of *ler* in DMEM.

The effect of DgcZ lacking the CZB domain (DgcZ-∆NT) on the LEE island is clear in another A/E pathogen, like EHEC. However, *C. rodentium* did not show this phenotype, indicating that the effect might be specific to regulatory components present in EPEC and EHEC but absent in *C. rodentium*.

DgcZ, which lacks the CZB domain (DgcZ-∆NT), is no longer active as a diguanylate cyclase. We propose that this second messenger is not directly responsible for activating virulence factors in EPEC and EHEC; instead, this phenotype might result from a protein-protein interaction between DgcZ lacking the CZB domain (DgcZ-∆NT) and an unknown regulator.

## Supplementary Material

fnag040_Supplemental_Files
