# Supplementary material for: Regulation of dgcZ in EPEC E2348/69 and the effect of partially deleting its CZB domain on the type III secretion system
Source: FEMS Microbiol Lett. 2026 Apr 15;373:fnag040. doi: 10.1093/femsle/fnag040 (PMC13131220; doi:10.1093/femsle/fnag040)
Supplement: fnag040_Supplemental_Files [file fnag040_supplemental_files.zip › Supplementary Table 1.docx]

Supplementary Table 1. Plasmids and strains used.

| Plasmid | Description | Reference |
| --- | --- | --- |
| pMPM T6 | Low copy number plasmid, Tc^R^, has an arabinose-inducible promoter | (Mayer, 1995) |
| pMPM T6-*dgcZ* | Derived from pMPM T6 that expresses DgcZ. | Previous work in the Laboratory |
| pMPM T6-*dgcZ* NdeI | Derived from pMPM T6-*dgcZ* that expresses DgcZ with part of its N-terminal domain truncated (DgcZ-∆NT). | In this work |
| pKK232-8 | Vector used to perform transcriptional fusions to the reporter gene *cat*.  Ap^R^ | Pharmacia LKB  Biotechnology |
| pCAT232 | Transcriptional fusion with the promoter region -232 to +76 of *bfpA* linked to the chloramphenicol acetyltransferase reporter gene. Ap^R^ | ((Puente et al., 1996)) |
| pKK232-8 *LEE1*-  cat | Transcriptional fusion of the region -162 to  +201 from the start of *LEE1* linked to the reporter gene chloramphenicol acetyl transferase. Ap^R^ | (Bustamante et al., 2001) |
| pKK232-8 *LEE2*- cat | Transcriptional fusion of the region -469 to +121 from the start of *LEE2* linked to the reporter gene chloramphenicol acetyl transferase. Ap^R^ | (Bustamante et al., 2001) |
| pKK232*dgcZa* | Transcriptional fusion of the region -214 to +28 from the start of *dgcZ* linked to the reporter gene chloramphenicol acetyl transferase. Ap^R^ | In this work |
| pKK232*dgcZb* | Transcriptional fusion of the region -214 to +2 from the start of *dgcZ* linked to the reporter gene chloramphenicol acetyl transferase. Ap^R^ | In this work |
| pKK232*dgcZc* | Transcriptional fusion of the region -75 to +28 from the start of *dgcZ* linked to the reporter gene chloramphenicol acetyl transferase. Ap^R^ | In this work |
| pKK232*dgcZd* | Transcriptional fusion of the región -75 to +2 from the start of *dgcZ* linked to the reporter gene chloramphenicol acetyl transferase. Ap^R^ | In this work |
| pMPMK3 | Plasmid that has an IPTG-inducible promoter, Km^R^ | (Mayer, 1995) |
| pMPMK3 - YgbI | Derived of pMPMK3 that express YgbI. | Previous work in the Laboratory |
| Strains | Description | Reference |
| *E. coli* MC4100 | *Escherichia coli* (strain K12 / MC4100) | (Oropeza et al., 2015) |
| *E. coli* - T6 | *E. coli* Sm^R^, transformed with pMPMT6, Tc^R^ | (Oropeza et al., 2015) |
| *E. coli* - dgcZ | *E. coli* transformed with pMPMT6 -  *dgcZ* | Previously work in the Laboratory |
| *E. coli* - dgcZ NdeI | *E. coli* transformed with pMPMT6 –  *dgcZ* NdeI | In this work |
| EPEC | WT, Sm^R^. EPECE2348/69 | (Levine et al., 1978) |
| EPEC - T6 | EPEC Sm^R^, transformed with pMPMT6, Tc^R^ | (Oropeza et al., 2015) |
| EPEC - *dgcZ* | EPEC transformed with pMPMT6 - *dgcZ* | Previous work in the Laboratory |
| EPEC - *dgcZ* NdeI | EPEC transformed with pMPMT6 –  *dgcZ* NdeI | In this work |
| Δ*ler* | EPEC mutant in *ler*. Sm^R^, Km^R^ | (Bustamante et al., 2001) |
| Δ*grlA* | EPEC mutant in *grlA*. Sm^R^, Km^R^ | (Bustamante et al., 2001) |
| Δ*perC* | EPEC mutant in *perC*. Sm^R^, Km^R^ | (Bustamante et al., 2001) |
| Δ*grlA* Δ*perC* | EPEC mutant in *grlA* y *perC*. Sm^R^, Km^R^ | (Bustamante et al., 2001) |
| -pEAF | EPEC without EAF plasmid. Sm^R^, Km^R^ | (Bustamante et al., 2001) |
| Δ*ler* - *dgcZ* NdeI | Δ*ler* transformed with pMPMT6 – *dgcZ*  NdeI | In this work |
| Δ*grlA* - *dgcZ* NdeI | Δ*grlA* transformed with pMPMT6 – *dgcZ*  NdeI | In this work |
| Δ*perC* - *dgcZ* NdeI | Δ*perC* transformed with pMPMT6 –  *dgcZ* NdeI | In this work |
| Δ*grlA* Δ*perC* - *dgcZ* NdeI | Δ*grlA* Δ*perC* transformed with pMPMT6 – *dgcZ* NdeI | In this work |
| -pEAF - *dgcZ* NdeI | -pEAF transformed with pMPMT6 – *dgcZ*  NdeI | In this work |
| EPEC-T6 - pCAT232 | EPEC-T6 transformed with pCAT232 | Previously work in the Laboratory |
| EPEC- *dgcZ* - pCAT232 | EPEC- *dgcZ* transformed with pCAT232 | Previously work in the Laboratory |
| EPEC- *dgcZ* NdeI - pCAT232 | EPEC- *dgcZ* NdeI transformed with pCAT232 | In this work |
| EPEC E2348/69 – p*LEE1* | EPEC transformed with pKK232-8 *LEE1*-cat | Previously work in the Laboratory |
| EPEC-T6 - p*LEE1* | EPEC-T6 transformed with pKK232-8 *LEE1*-cat | Previously work in the Laboratory |
| EPEC- *dgcZ* - p*LEE1* | EPEC- *dgcZ* transformed with pKK232-8  *LEE1* | Previously work in the Laboratory |
| EPEC- *dgcZ* NdeI - p*LEE1* | EPEC- *dgcZ* NdeI transformed with pKK232-8 *LEE1*-cat | In this work |
| EPEC E2348/69 – p*LEE2* | EPEC transformed with pKK232-8 *LEE2*-cat | Previously work in the Laboratory |
| EPEC-T6 – p*LEE2* | EPEC-T6 transformed with pKK232-8 *LEE2*-cat | Previously work in the Laboratory |
| EPEC- *dgcZ* – p*LEE2* | EPEC- *dgcZ* transformed with pKK232-8  *LEE2*-cat | Previously work in the Laboratory |
| EPEC- *dgcZ* NdeI - p*LEE2* | EPEC- *dgcZ* NdeI transformed with pKK232-8 *LEE2*-cat | In this work |
| EPEC - *dgcZa* | EPEC transformed with pKK232*dgcZa* | In this work |
| EPEC - *dgcZb* | EPEC transformed with pKK232*dgcZb* | In this work |
| EPEC - *dgcZc* | EPEC transformed with pKK232*dgcZc* | In this work |
| EPEC - *dgcZd* | EPEC transformed with pKK232*dgcZd* | In this work |
| EPEC – *dgcZb* – K3 | EPEC – *dgcZb* transformed with pMPMK3 | In this work |
| EPEC – *dgcZb* – *YgbI* | EPEC – *dgcZb* transformed with pMPMK3 - *YgbI* | In this work |
| EPEC – *dgcZd* – K3 | EPEC – *dgcZd* transformed with pMPMK3 | In this work |
| EPEC – *dgcZd* – *YgbI* | EPEC – dgcZd transformed with pMPMK3 - *YgbI* | In this work |
