# Supplementary material for: Regulation of dgcZ in EPEC E2348/69 and the effect of partially deleting its CZB domain on the type III secretion system
Source: FEMS Microbiol Lett. 2026 Apr 15;373:fnag040. doi: 10.1093/femsle/fnag040 (PMC13131220; doi:10.1093/femsle/fnag040)
Supplement: fnag040_Supplemental_Files [file fnag040_supplemental_files.zip › Supplementary Table 2.docx]

Supplementary Table 2. Oligonucleotides used to amplify DNA fragments from the regulatory region of *dgcZ*.

| Oligonucleotide | Sequence5´🡪 3´ |
| --- | --- |
| dgcZBamFor **BamHI** | GCGGGATCCGCCTGATGGCCTGAACTACT |
| dgcZCysBFor **BamHI** | GCGGGATCCCTCAGAGATGCTCATAACTCCG |
| dgcZHindRev **HindIII** | CGCAAGCTTGCCACTCCTTTTTCACAGTTCC |
| dgcZcsrARev **HindIII** | CGCAAGCTTGCGCGCTATTCTAACGAGAGAA |
| dgcZcysB1Bam **BamHI** | GCGGGATCCGGGTTATGCTATTTGTCATCCT |
| dgcZcysB2Bam **BamHI** | GCGGGATCCGCTATTTGTCATCCTTTTAATTGCG |
| dgcZcysB3Bam **BamHI** | GCGGGATCCCCTTTTAATTGCGATAATGCAGAG |
