# Supplementary figures and images for: Regulation of dgcZ in EPEC E2348/69 and the effect of partially deleting its CZB domain on the type III secretion system
Source: FEMS Microbiol Lett. 2026 Apr 15;373:fnag040. doi: 10.1093/femsle/fnag040 (PMC13131220; doi:10.1093/femsle/fnag040)

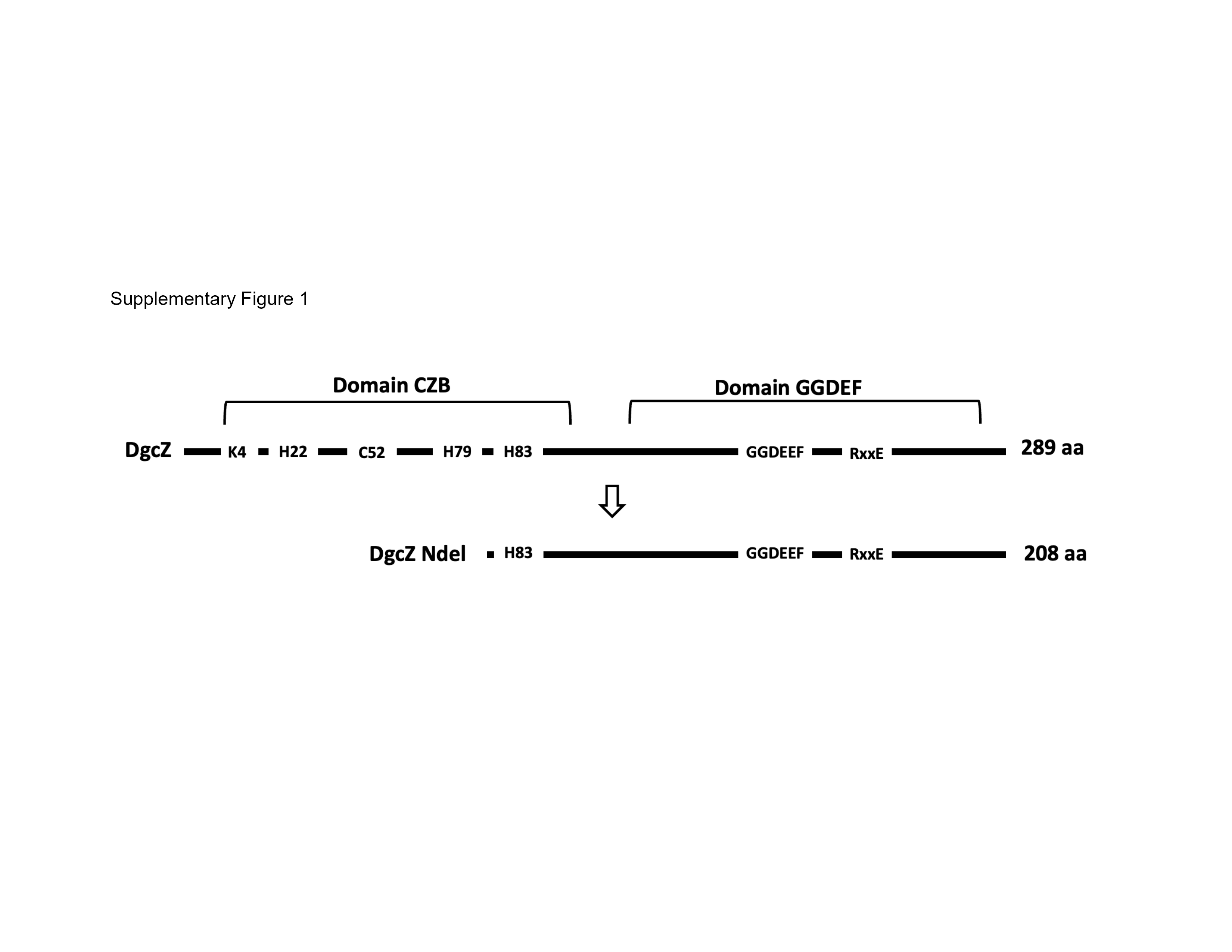

Supplement: fnag040_Supplemental_Files [file fnag040_supplemental_files.zip › Supplementary Figure 1.tiff]

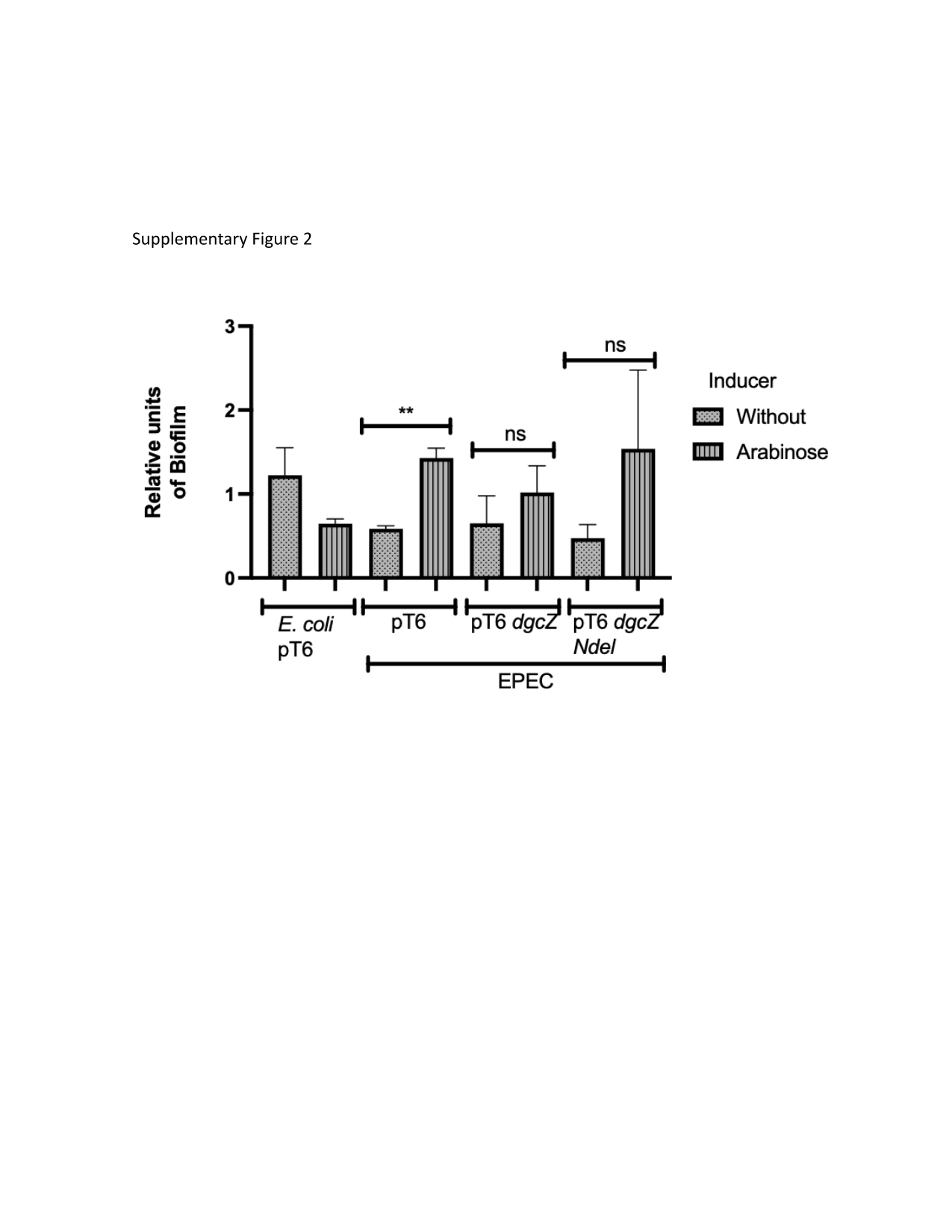

Supplement: fnag040_Supplemental_Files [file fnag040_supplemental_files.zip › Supplementary Figure 2.tiff]

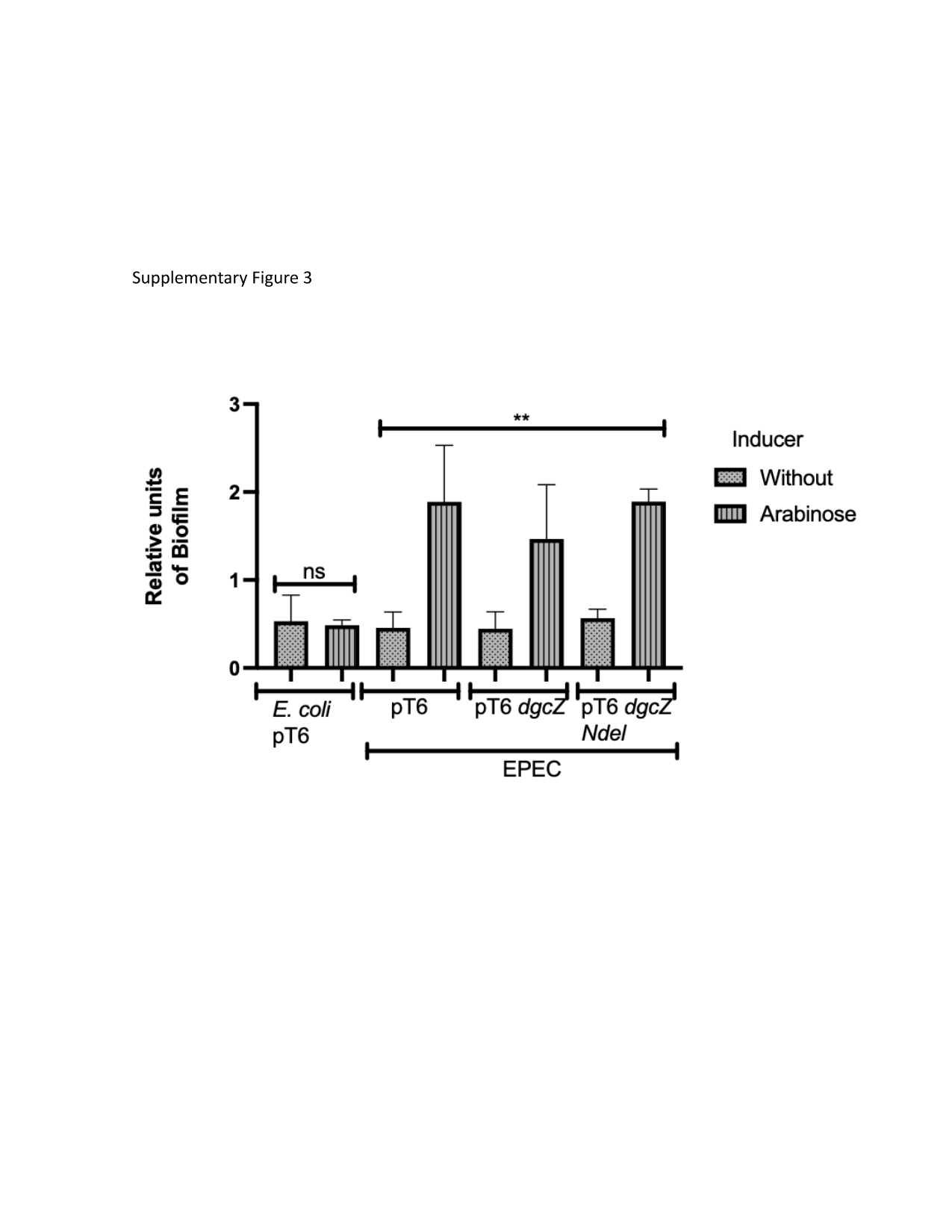

Supplement: fnag040_Supplemental_Files [file fnag040_supplemental_files.zip › Supplementary Figure 3.tiff]

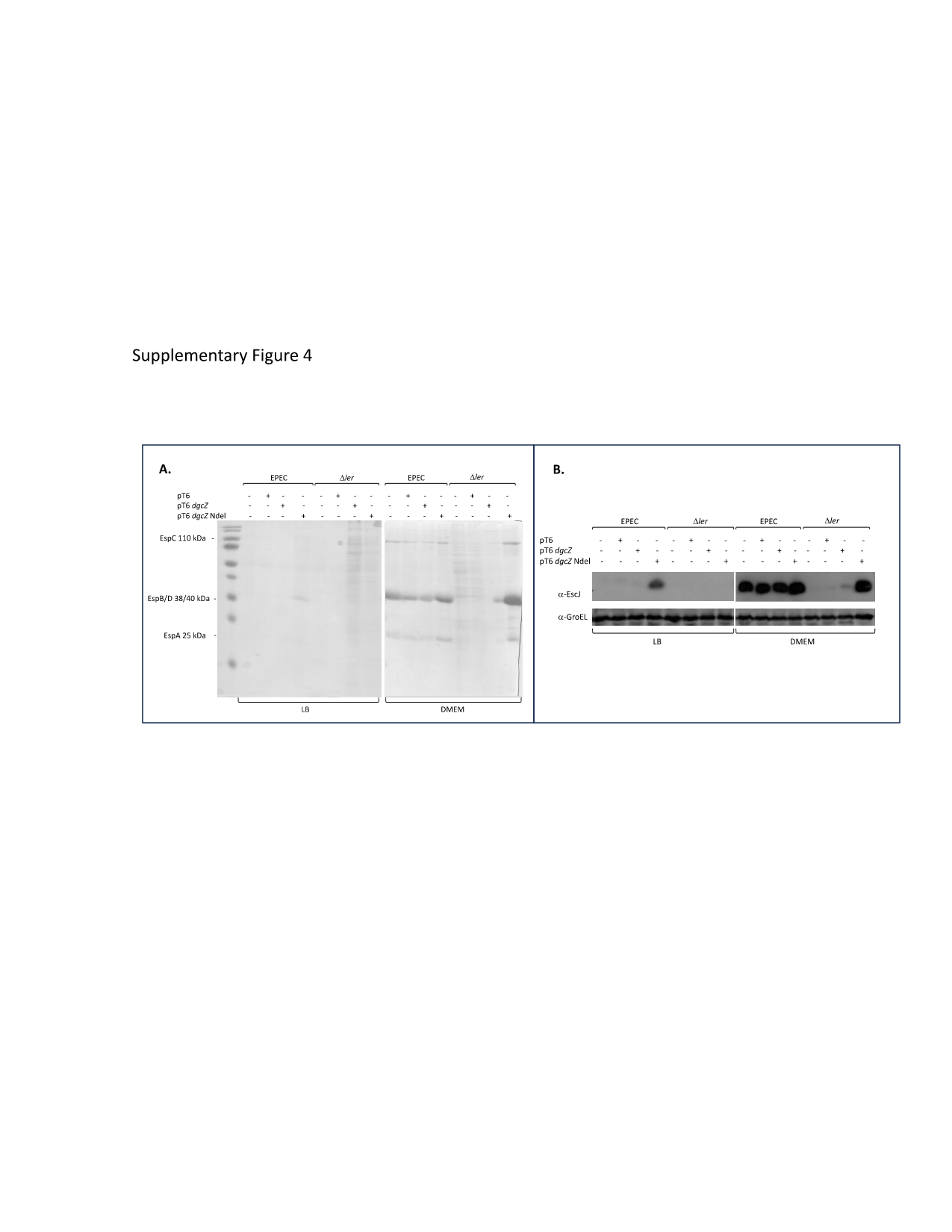

Supplement: fnag040_Supplemental_Files [file fnag040_supplemental_files.zip › Supplementary Figure 4.tiff]
